# A Challenging Case of Severe Dysphagia in Multiple System Atrophy

**DOI:** 10.1007/s00455-025-10871-x

**Published:** 2025-08-15

**Authors:** Miguel Limbert Ramos, Rumi Ueha, Takao Goto, Kentaro Ichijo, Hirofumi Sugita, Yuichiro Shirota, Kenji Kondo

**Affiliations:** 1 Department of Otolaryngology and Head and Neck Surgery, The University of Tokyo, Tokyo, Japan; 2 Department of Otolaryngology– Head and Neck Surgery, Bataan General Hospital and Medical Center, Balanga City, Bataan, Philippines; 3 Swallowing Center, The University of Tokyo Hospital, 7-3-1 Hongo, Bunkyo-ku, Tokyo, 113-8655 Japan; 4 Department of Neurology, The University of Tokyo, Tokyo, Japan

**Keywords:** Laryngeal suspension, Swallowing improvement surgery, Dysphagia, Multiple system atrophy, High-resolution pharyngeal manometry

## Case Presentation

**Fig. 1:**
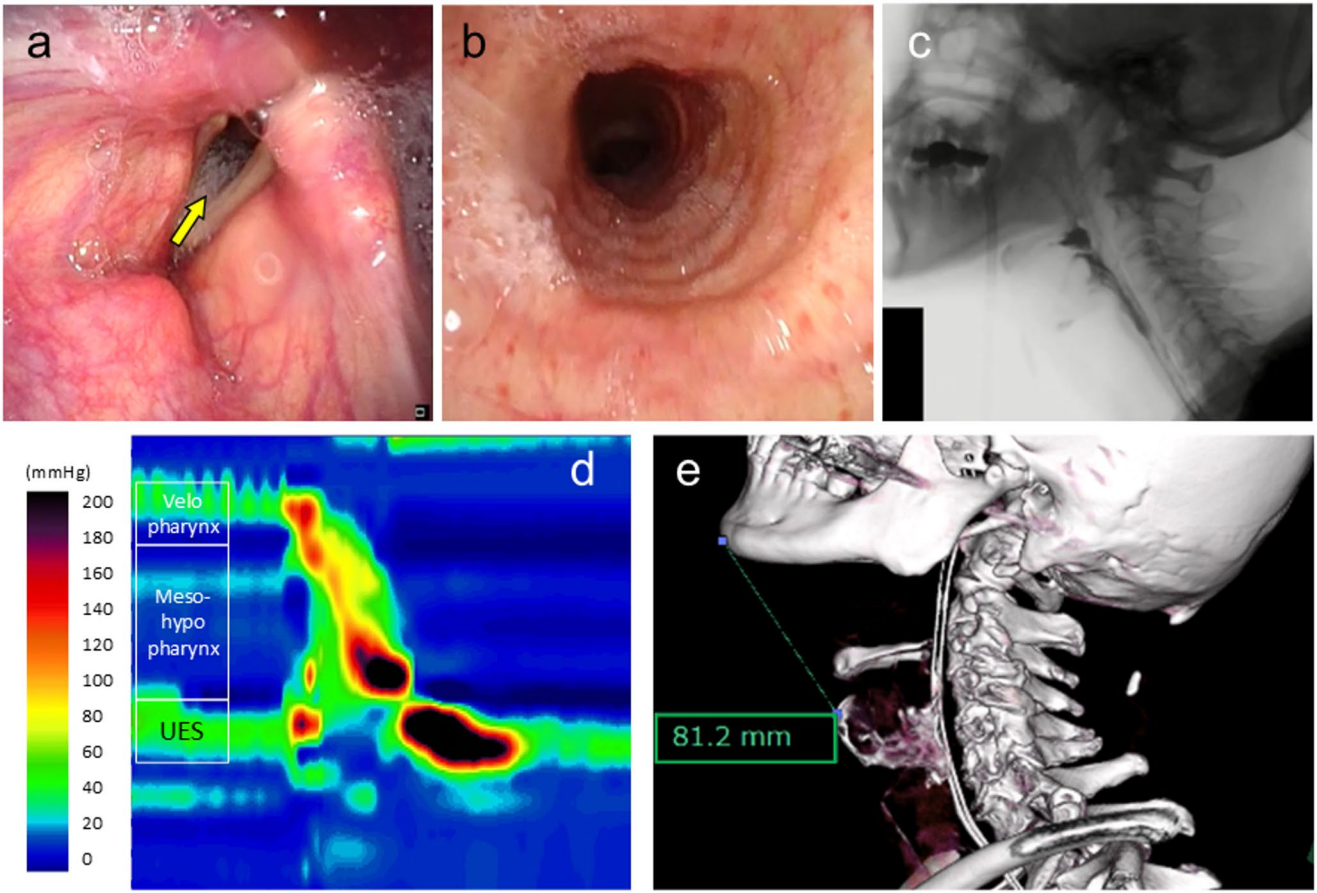
Preoperative examinations. (a, b) laryngoscopy revealed saliva pooling in the hypopharynx and entry of saliva into the trachea (yellow arrow). (c) videoflouroscopic swallowing study with 5 ml thickened contrast agent showed silent aspiration into the airway. (d) high-resolution manometry (HRM) tracing obtained during swallowing. (e) cervical computed tomography scan showed the preoperative thyromental distance of 81.2 mm. The potential extent of elevation and anteriorization of the larynx, as it approximates the mandible, can also be estimated during the planning process. UES: upper esophageal sphincter

A 70-year-old male with a 9-year history of multiple system atrophy-cerebellar variant (MSA-C), initially presenting with dizziness and slurred speech with symptoms of swallowing dysfunction that gradually developed over time. For the past 6 months, the patient’s overall condition progressively declined marked by recurrent episodes of aspiration pneumonia and urinary tract infection. During another onset of aspiration pneumonia, the patient was admitted under the neurology department of our institution. As the frequency of suctioning of secretions had become an increasing concern (approximately once every hour), he was referred to the otolaryngology department for evaluation of swallowing function and to discuss the potential indications for tracheostomy. On physical examination, he exhibited severe dysarthria, reduced tongue mobility, impaired pharyngeal sensation, marked cervical rigidity preventing neck flexion, and impaired laryngeal elevation. Laryngoscopic examination revealed intact velopharyngeal closure, involuntary movement of bilateral vocal folds, decreased laryngeal sensation, saliva pooling in the hypopharynx, and aspiration into the trachea (Fig. 1-a, b). Videofluoroscopic swallowing study (VFSS) using 5mL of thickened contrast agent (100 mPa·s) showed weak pharyngeal contraction, poor laryngeal anterior movement at maximum elevation, and silent aspiration of the contrast agent into the airway (Fig. 1-c). High-resolution manometry (HRM) was performed, with the findings shown in Fig. 1-d. A cervical computed tomography (CT) scan revealed a low-lying larynx, with a measured thyromental distance of 81.2 mm (Fig. 1-e). Given the constellation of findings which include persistent aspiration, impaired laryngeal elevation, and the HRM tracing, what would be the most appropriate management strategy for the patient?

**What is the underlying pathophysiology of dysphagia**,** and what are the corresponding therapeutic approaches?**

**Fig. 2:**
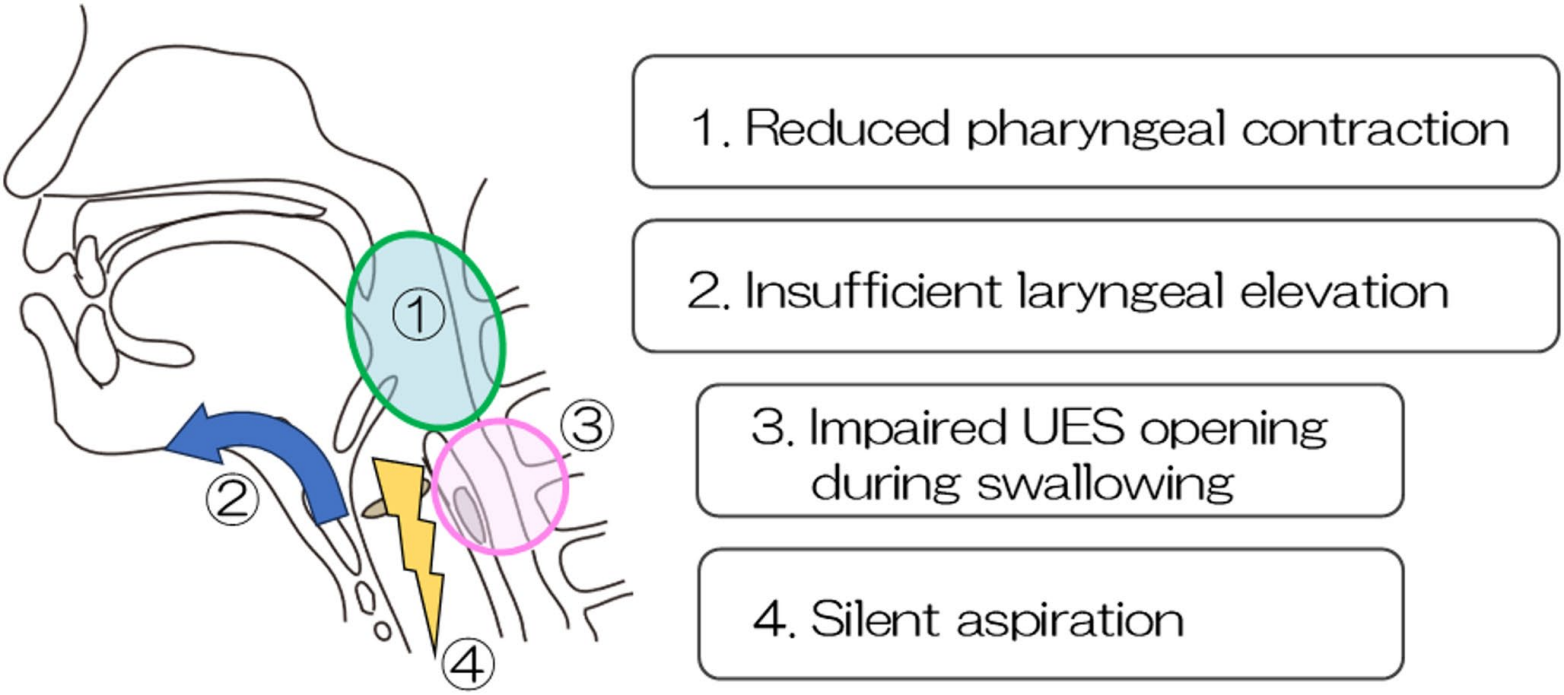
Pathophysiology of dysphagia. Diagram illustrating key dysfunctions: weakened pharyngeal contraction, limited laryngeal elevation, compromised UES opening, and silent aspiration. UES: upper esophageal sphincter

Based on the results of all the tests performed, it was determined that the progression of multiple system atrophy (MSA) had led to a decline in both sensory and motor functions of swallowing. This included reduced pharyngeal contraction force, impaired laryngeal elevation, and silent aspiration. Regarding upper esophageal sphincter (UES) function, it was speculated that impaired (UES) opening during swallowing was attributable to insufficient anterior–superior hyolaryngeal movement, as UES relaxation was preserved to a certain extent on HRM (Fig. 1-d). In summary, although the patient retained some ability to transfer food orally to some extent, the reduced pharyngeal pressure around the tongue base and inadequate laryngeal elevation resulted in insufficient UES opening. This led to difficulty in bolus passage into the esophagus and partial silent aspiration. As the disease progresses, further deterioration of swallowing function is anticipated (Fig. 2).

Given the repeated episodes of pneumonia caused by silent aspiration, a tracheostomy was considered essential to ensure airway protection and to provide a route for suctioning. However, it was predicted that the tracheostomy could further compromise swallowing function, making oral intake more challenging. Furthermore, even with a tracheostomy, the use of a speech-type tracheostomy tube was deemed infeasible due to the persistent silent aspiration. Considering the progressive nature of MSA and the need for effective secretion management via tracheostomy, additional interventions beyond tracheostomy were considered necessary to support oral intake and reduce the frequency of suctioning.

**How should treatment options be individualized for severe dysphagia in MSA?**

**Fig. 3:**
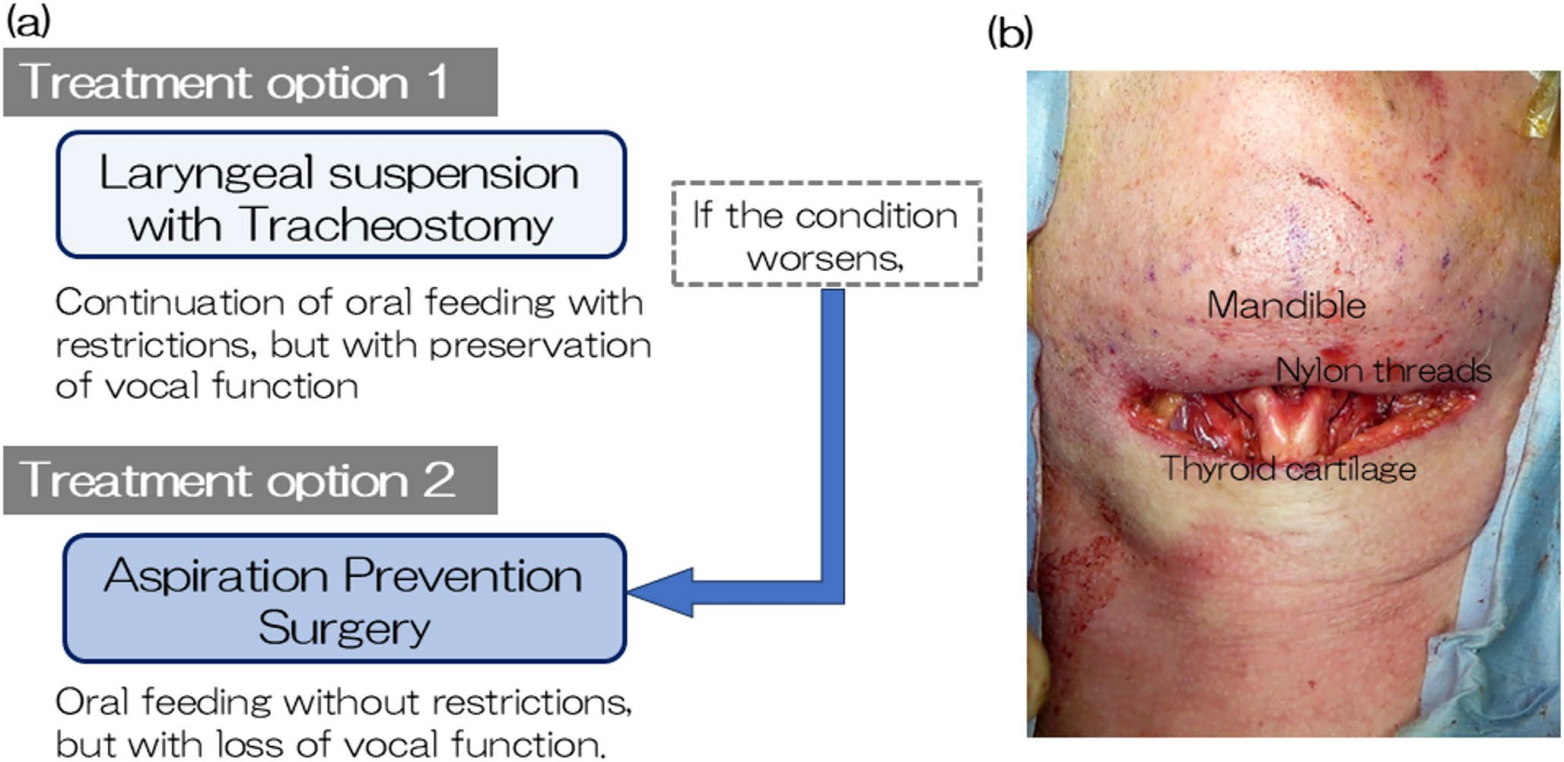
Treatment options and intraoperative view. (a) clinical decision flowchart comparing two surgical options: laryngeal suspension with tracheostomy, and aspiration prevention surgery. (b) intraoperative view of thyroid cartilage elevated and anchored to the mandible with nylon threads

Taking into account the patient’s swallowing dynamics, cricopharyngeal myotomy was deemed less effective, whereas laryngeal suspension was expected to improve swallowing by enhancing UES opening.　Based on the patient’s condition, the two main therapeutic options considered were: (1) laryngeal suspension surgery with tracheostomy, and (2) aspiration prevention surgery. The former option could improve swallowing function while preserving vocal function, however, it was not expected to fully prevent aspiration. Moreover, with the anticipated progression of MSA, both oral intake and phonation were likely to become compromised. In contrast, aspiration prevention surgery would eliminate the risk of aspiration and allow continued oral intake, but at the cost of vocal function. The surgical invasiveness would be similar in both procedures. If laryngeal suspension is chosen, aspiration prevention surgery would remain a feasible option should the disease progress (Fig. 3-a).

Following a comprehensive discussion of the available surgical options, the patient chose to undergo laryngeal suspension, expressing a desire to maintain vocal function for as long as possible and to continue oral intake, even if only for a limited time.　The surgery was performed under general anesthesia. The mandible and thyroid cartilage were sutured together using nylon threads (size 2) to reduce the thyromental distance (thyro-mandibular suspension) (Fig. 3-b). After placement of a single negative-pressure drain in the surgical site, the wound was closed. A tracheostomy was performed and a cuffed tracheostomy tube (ID 7.5 mm) was inserted to secure the airway and suction route.

Postoperative nutritional management was initiated with nasogastric feeding. The drain was removed on postoperative day (POD) 4 without any complications. As tracheal suctioning volume decreased and surgical site swelling subsided, the tracheostomy tube was replaced with a cuffed speech-type tube. Oral intake of jelly diet began on POD 6, with careful adjustment of patient positioning, food consistency, and bolus volume (5mL).

**Fig. 4:**
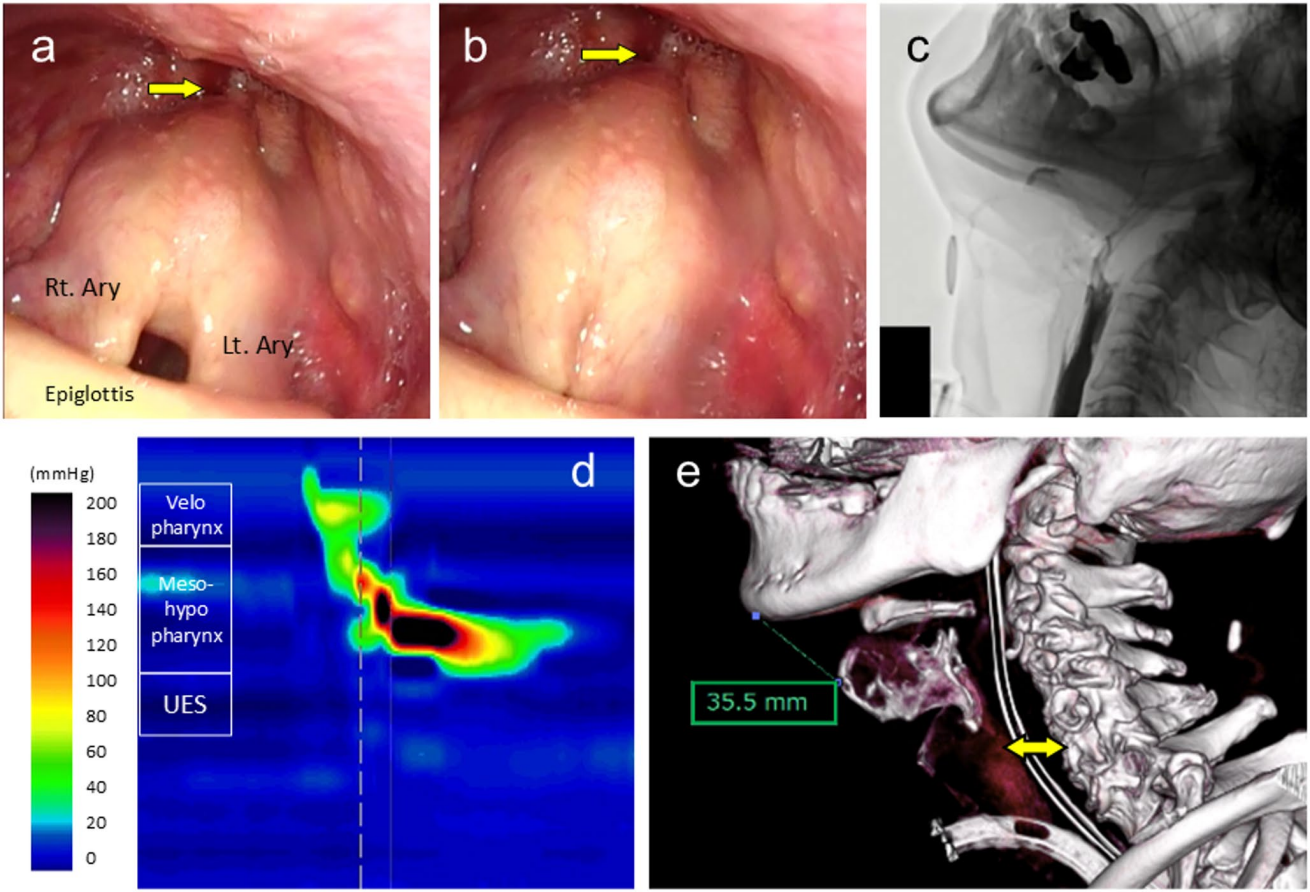
Postoperative examinations. (a, b) anterior-superior traction of the bilateral arytenoid cartilages (Ary) and visualization of the upper esophageal sphincter (UES, yellow arrows) during inspiration (a) and phonation (b). (c) videoflouroscopic swallowing study using 5 mL of thin liquid contrast agent at 60° postural angle. (d) postoperative high-resolution manometry revealed sustained opening of the UES. (e) cervical CT scan showed anterior-superior displacement of the thyroid cartilage and reduced thyromental distance (35.5 mm), with widened UES opening (double-headed yellow arrow)

Postoperative laryngoscopy showed anterior-superior traction of bilateral arytenoid cartilages and significant opening of the UES upon swallowing, with minimal to no pooling of secretions at the pyriform sinus (Fig. 4-a, b). On POD 11, VFSS showed that 5 mL of mildly thickened contrast agent passed smoothly through the UES, with no evidence of pharyngeal residue or aspiration at a 60° upright position. The patient’s diet was upgraded from jelly consistency to a blenderized texture.

A gastrostomy tube was placed on POD 20 to secure a nutritional route for possible future use. A repeat VFSS was performed on postoperative day 34, prior to discharge, demonstrated significant improvement in bolus passage with 5 mL of thin liquid contrast agent, without evidence of retention or aspiration (Fig. 4-c). HRM confirmed sustained relaxation of the UES at rest and during swallowing (Fig. 4-d). Postoperative cervical CT revealed a shortened thyromental distance of 35.5 mm (Fig. 4-e).

At three months postoperatively, the patient remained on a speech cannula and had progressed to a minced and moist diet, representing a significant improvement in swallowing function compared to his preoperative status.

## Discussion

Dysphagia in patients with neurological disorders presents a complex therapeutic challenge. As the disease progresses and conservative management becomes insufficient, treatment strategies should be individualized to fit the patient’s current medical condition and personal preferences. The present case highlights the potential role of laryngeal elevation surgery in improving swallowing function while preserving vocal function in a patient with severe dysphagia due to multiple system atrophy (MSA).

Current management of dysphagia in neurological disorders primarily centers on compensatory strategies, including dietary modifications and adapted feeding techniques [1–3]. In cases of severe dysphagia, aspiration prevention surgeries have been employed, particularly in patients with progressive neurological disorders such as Parkinson’s disease (PD) [4], multiple system atrophy (MSA) [5, 6], and amyotrophic lateral sclerosis (ALS) [4, 5, 7–10]. However, aspiration prevention surgeries come at the cost of permanently sacrificing vocal function which significantly impact the patient’s quality of life (QOL), especially in individuals who prioritize communication.

In contrast, swallowing improvement surgeries are designed to restructure the laryngeal anatomy to promote improved passage of bolus while reducing the risk of aspiration and preserving phonation [11]. Although patients with advanced neurological disorders are often considered unsuitable candidates due to the progressive nature of their conditions, there are cases where such interventions may offer temporary, yet significant improvements in swallowing function and QOL [2].　Surgical techniques to improve swallowing such as cricopharyngeal myotomy (CPM) have been performed in patients with Parkinson’s disease (PD) [12–14] and inclusion body myositis [15–19]. In patients with PD and Parkinson-related disorders, CPM and balloon dilatation have shown efficacy in addressing UES passage disorders [12–14, 20]. Table 1 summarizes the literature on swallowing improvement surgeries in patients with neurodegenerative disorders over the past three decades. CPM is effective in selected patients with impaired UES relaxation, as it relieves functional obstruction, promotes bolus passage through the hypopharynx, and reduces the risk of aspiration. However, it is important to recognize that the progression of swallowing symptoms in neurological disorders comes at different stages.

**Table 1:** List of studies on swallowing improvement surgeries done on patients with neurological disorders

Study	Year Published	No. of Subjects	Condition/s	Intervention	Outcome
*Wintzen et al.* [17]	1988	4	Inclusion Body Myositis	Cricopharyngeal Myotomy	3 out of 4 subjects had improved signs and symptom of dysphagia and aspiration
*Born et al.* [12]	1996	4	Parkinson’s Disease	Cricopharyngeal Myotomy	4 out of 4 Subject have experienced excellent and sustained relief of esophageal symptoms following surgery.
*Poirier et al.* [13]	1997	2 1 7	Amyotrophic Lateral Sclerosis Parkinson’s Disease Pseudobulbar Palsy	Cricopharyngeal Myotomy	No improvement post-surgery in ALS and PD. 6 out of 7 subjects with PP had relief of symptoms.
*Houser*,* S. M.*,* Calabrese*,* L. H.*,* & Strome*,* M.* [19]	1998	2	Inclusion Body Myositis	Cricopharyngeal Myotomy	2 out of 2 subjects noted significant improvement in swallowing function
*Hoesseini et al.* [16]	2016	5 3 1 1 4	Oculopharyngeal Muscular Dystrophy Inclusion Body Myositis Mitochondrial Myopathy Spinal Muscle Atrophy Others	Cricopharyngeal Myotomy	12 out of 14 subjects reported subjective relief of symptoms after the 1 st surgery.
*McMillan et al.* [18]	2021	41	Inclusion Body Myositis	Cricopharyngeal Myotomy	11 patients had recurrence of dysphagia
*Wu et al.* [14]	2021	8	Parkinson’s Disease	Cricopharyngeal Myotomy	7 out of 8 subjects appreciated improvement in swallowing after 1 month.
*Shenoy et al.* [22]	2023	1	Lateral Medullary Syndrome (Wallenberg)	Cricopharyngeal Myotomy	Noted improvement of symptoms 1 week after the procedure.

The case presented demonstrated classic features of MSA which includes impaired laryngeal elevation, poor pharyngeal contraction, and silent aspiration [20]. However, HRM revealed preserved UES relaxation, suggesting that the principal barrier to bolus passage was mechanical, rather than functional failure of the sphincter. This finding helped us in surgical decision-making process, particularly in ruling out CPM as an intervention, which would have been inappropriate given the intact UES relaxation. This case also emphasizes the importance of comprehensive preoperative evaluation, including VFSS and HRM, to tailor surgical interventions appropriately.

Laryngeal suspension surgery was performed to mechanically shorten the thyromental distance and advance the larynx anteriorly. This anterior-superior repositioning facilitates epiglottic retroflexion and promotes effective UES opening during swallowing [11, 21]. This intervention enabled the patient to resume oral intake while preserving vocal function, aligning with the patient’s preferences and contributing to overall improvement in QOL. However, due to the progressive nature of MSA, the long-term efficacy of this approach is inherently limited. As the disease advances, further deterioration in swallowing function and increased aspiration risk are expected. When this occurs, aspiration prevention surgery should be considered as a feasible next step.

To our knowledge, this is the first documented case where isolated laryngeal elevation surgery, rather than aspiration prevention surgery or CPM, has led to measurable improvement in swallowing function in patients with MSA. While this report adds to the limited but growing body of literature on surgical options for dysphagia in neurological disorders, further studies are warranted to establish patient selection criteria, optimal timing, and evaluate long-term outcomes. A multidisciplinary approach involving otolaryngology, neurology, and rehabilitation teams is essential for identifying patients who may benefit from this type of surgery.

## Data Availability

Data are available on a reasonable request.
